# Molecular study of metallo-β-lactamases and integrons in *Acinetobacter baumannii* isolates from burn patients

**DOI:** 10.1186/s12879-021-06513-w

**Published:** 2021-08-09

**Authors:** Mahnaz Nikibakhsh, Farzaneh Firoozeh, Farzad Badmasti, Kourosh Kabir, Mohammad Zibaei

**Affiliations:** 1 Department of Microbiology, School of Medicine, Alborz University of Medical Sciences, P.O. Box: 3149779453, Karaj, Iran; 2 Evidence-Based Phytotherapy and Complementary Medicine Research Center, Alborz University of Medical Sciences, Karaj, Iran; 3 Department of Bacteriology, Pasteur Institute of Iran, Tehran, Iran; 4 Department of Community Medicine and Epidemiology, School of Medicine, Alborz University of Medical Sciences, Karaj, Iran; 5 Department of Parasitology and Mycology, School of Medicine, Alborz University of Medical Sciences, Karaj, Iran

**Keywords:** *Acinetobacter baumannii*, Metallo-β-lactamases, Integrons, Gene cassettes, Burn, *bla*_VIM_, *bla*_IMP_

## Abstract

**Background:**

Productions of metallo-β-lactamases enzymes are the most common mechanism of antibiotic resistance to all beta-lactam classes (except monobactams) in *Acinetobacter baumannii*. MBLs are usually associated with gene cassettes of integrons and spread easily among bacteria. The current study was performed to detect the genes encoding MBLs and integron structures in *A. baumannii* isolates from burn patients.

**Methods:**

This study was performed on 106 non-duplicate *A. baumannii* isolates from burn patients referred to Shahid Motahari Hospital in Tehran. Antibiotic susceptibility of *A. baumannii* isolates was performed using disk diffusion and broth microdilution method in accordance with the CLSI guidelines. The presence of class 1 integron and associated gene cassettes as well as MBLs-encoding genes including *bla*_VIM_, and *bla*_IMP_ were investigated using PCR and sequencing techniques.

**Results:**

In this cross-sectional study all (100%) of the *A. baumannii* isolates examined were multidrug resistant. All isolates were sensitive to colistin and simultaneously all were resistant to imipenem. PCR assays showed the presence of *bla*_VIM_ and *bla*_IMP_ genes in 102 (96.2%) and 62 (58.5%) isolates of *A. baumannii* respectively. In addition, 62 (58.5%) of the *A. baumannii* isolates carried integron class 1, of which 49 (79.0%) were identified with at least one gene cassette. Three types of integron class 1 gene cassettes were identified including: *arr2*, *cmlA5*, *qacE1* (2300 bp); *arr-2*, *ereC*, *aadA1*, *cmlA7*, *qacE1* (4800 bp); and *aac(3)-Ic*, *cmlA5* (2250 bp).

**Conclusion:**

A high prevalence of MBLs genes, especially *bla*_VIM_, was identified in the studied MDR *A. baumannii* isolates. In addition, most of the strains carried class 1 integrons. Furthermore, the gene cassettes arrays of integrons including *cmlA5* and *cmlA7* were detected, for the first time, in *A. baumannii* strains in Iran.

## Background

Burn infections are a noticeable health problem, especially in developing countries [1]. It has been documented that about 75% of death in patients with burn injuries are due to infections [2] *Acinetobacter baumannii* is one of the most common causes of nosocomial infections with high mortality and morbidity among hospitalized patients, especially in burn and intensive care units [3, 4]. Nowadays, the emergence and spread of antibiotic resistance in *A. baumannii* is a major global challenge [4]. Carbapenems are considered drugs of choice for the treatment of severe infections caused by MDR-*A. baumannii* [5]. Unfortunately, carbapenem resistance is increasing among *A. baumannii* isolates, which is alarming [5].

Various mechanisms are involved in the development of carbapenem resistance in *A. baumannii* including β-lactamases acquisition, outer membrane proteins and PBPs alteration, overexpression of efflux pumps and gene mutation of CarO [6].

One of the most important mechanisms of antibiotic resistance in *A. baumannii* is the production of β-lactamase enzymes, the genes of which are usually carried on mobile genetic elements, including integrons [7]. Beta-lactamases are grouped into four classes based on the amino acid sequence, including: A, B, C, and D [8]. Resistance to carbapenems is usually dependent on β-lactamases of class B (MBLs) and D (OXA-type carbapenemases) [9]. OXA β-lactamases or OXA-type carbapenemases, include distinct subgroups from which OXA-23-like, OXA-24-like, OXA-40-like, OXA-51-like, OXA-58-like and OXA-143-like have been found in *A. baumannii* strains [9, 10]. MBLs families are more important than other β-lactamases, due to their ability to hydrolyze a wide range of β-lactam antibiotics, especially carbapenems [11, 12]. Several MBLs including VIM and IMP have been identified among *A. baumannii* strains [13, 14]. Different IMP–type enzymes have been described in the globe among Gram-negative bacilli especially Enterobacterales and non-fermenter organisms including *Acinetobacter* spp. [15]. Previous studies have shown that the prevalence of MBL-producing strains of *A. baumannii* in the world is increasing, although there are different reports in various geographical areas [13].

*Acinetobacter* has a high potential for the acquisition of resistance genes through mobile genetic elements, including integrons [15]. MBLs are generally encoded on the gene cassettes of class 1 integrons and spread readily among *A. baumannii* strains [16]. The presence of integrons and association with genes encoding MBLs are frequently reported in *A. baumannii* isolates [17].

The current study was performed to detect the genes encoding MBLs and class 1 integron structures in *A. baumannii* isolates from burn patients.

## Methods

### Ethical consideration

Informed consent was obtained from all subjects, and all methods were carried out in accordance with the relevant guidelines and regulations of Ethics Clearance Committee of the Alborz University of Medical Sciences.

### Bacterial isolates and identification

The current study was conducted between December 2019 and November 2020. A total of 106 non-duplicate *A. baumannii* isolates were collected from hospitalized burn patients at Shahid Motahari Hospital in Tehran, Iran. The collected clinical isolates were transferred to the laboratory of the Department of Microbiology, School of Medicine, Alborz University of Medical Sciences. Standard biochemical tests were used to identify the Gram-negative bacilli isolates as *A. baumannii* strains These tests included catalase, oxidase, O/F test, motility, citrate utilization test and growth on TSI agar (Merck, Germany) [18]. The diagnosed* A. baumannii* strains were cultured in TSB (Merck, Germany) supplemented with 20% glycerol and stored at − 20 °C for further studies. The phenotypically isolated *A. baumannii* strains were confirmed by PCR and sequencing of the *rpo*B gene [19]. The *A. baumannii* ATCC 19606 was used as a control strain.

### Antibiotic susceptibility testing

Susceptibility of *A. baumannii* to imipenem (10 µg), gentamicin (10 µg), ciprofloxacin (5 µg), ampicillin-sulbactam (20 µg), trimethoprim/sulfamethaxazole (1.25/23.75 μg), ceftazidime (30 µg), doxycycline (30 µg), and minocycline (30 μg) was performed by disk diffusion method accordant with CLSI standard guidelines [20]. The studied antibiotics were purchased from MAST Company (Mast, UK). The MICs of imipenem [breakpoints (μg/ml): susceptible: ≤ 2; intermediate: 4; resistant: ≥ 8], and colistin [breakpoints (μg/ml): susceptible: ≤ 2; intermediate: -; resistant: ≥ 4] were determined by the broth microdilution method according to the guidelines of the CLSI [20]. The quality control strain was *Escherichia coli* ATCC 25922. The bacteria were categorized to MDR, XDR or PDR based on Magiorakos et al., [21] criteria.

### Determination of integrons and associated gene cassettes

Extraction of genomic DNA of *A. baumannii* strains was performed using boiling method [22]. The presence of integrons class 1 and related gene cassettes were determined by PCR using related primers [23]. The gene cassettes of integrons were amplified using primers and PCR conditions as described previously [23, 24]. Amplified gene cassettes were sent for sequencing (Macrogen Research, Seoul, Korea). The sequences obtained were compared with those deposited in the NCBI database with using BLAST program (http://blast.ncbi.nlm.nih.gov/Blast.cgi) to determine the cassettes arrays of integrons.

### Molecular detection of metallo-β-lactamases-encoding genes

The amplification of the metallo-β-lactamases-encoding genes including *bla*_VIM_, and *bla*_IMP_ which is associated with carbapenem resistance was performed by PCR assays in all isolates. The relevant primers used for PCR testing are listed in Table 1. The PCR conditions were performed according to the protocol provided [22].

**Table 1:** Primers used for PCR assays in this study

Gene	Primer	Sequence (5′–3′)	Product (bp)	References
*intI1*^†^	F	CAG TGG ACA TAA GCC TGT TC	160	[23]
	R	CCC GAG GCA TAG ACT GTA		
*CS*^††^	F	GGC ATC CAA GCA GCA AG	Variable	[23, 24]
	R	AAG CAG ACT TGA CCT GA		
*bla*_VIM_	F	GAT GGT GTT TGG TCG CAT A	390	[22]
	R	CGA ATG CGC AGC ACC AG		
*bla*_IMP_	F	GGA ATA GAG TGG CTT AAY TCT C	232	[22]
	R	GGT TTA AYA AAA CAA CCA CC		

^†^*Intl1*: Class 1integron-integrase gene

^††^*CS*: Conserved segment of class 1 integrons

### Statistical analysis

All statistical analysis was performed using SPSS software version 21 (SPSS, Inc.). The differences between variables were evaluated by the chi-square (χ^2^) test and *P-*values (*P* < 0.05) were interpreted statistically significant.

## Results

In this cross-sectional study, a total of 106 non-duplicate *A. baumannii* isolates were collected from burn wounds of hospitalized patients. Seventy-six of the *A. baumannii* strains were isolated from male (71.7%) and 30 females (28.3%) patients. According to the results of antimicrobial susceptibility testing, all (100%) of our *A. baumannii* isolates were identified as MDR and 101 (95.3%) were XDR**,** however, PDR phenotype were not detected in any of the isolates. The antibiotic susceptibility profiles of the *A. baumannii* isolates by disk diffusion method are shown in Table 2. Determination of MICs of imipenem and colistin showed that all isolates were sensitive to colistin (MIC of ≤ 2 μg/ml) and at the same time all were resistant to imipenem (MIC of ≥ 8 μg/ml).

**Table 2:** Antibiotic susceptibility of *Acinetobacter baumannii* isolates by disk diffusion method

Antibiotic	Susceptible (S), N (%)	Intermediate (I), N (%)	Resistant (R), N (%)	Interpretive categories and zone diameter breakpoints (mm)		
				S	I	R
Imipenem	0 (0.0)	0 (0.0)	106 (100)	≥ 22	19–21	≤ 18
Ceftazidime	0 (0.0)	0 (0.0)	106 (100)	≥ 18	15–17	≤ 14
Gentamicin	5 (4.7)	2 (1.9)	99 (93.4)	≥ 15	13–14	≤ 12
Doxycycline	101 (95.3)	0 (0.0)	5 (4.7)	≥ 13	10–12	≤ 9
Minocycline	102 (96.2)	4 (3.8)	0 (0.0)	≥ 16	13–15	≤ 12
Ciprofloxacin	5 (4.7)	0 (0.0)	101 (95.3)	≥ 21	16–20	≤ 15
Ampicillin-sulbactam	100 (94.3)	0 (0.0)	6 (5.7)	≥ 15	12–14	≤ 11
Trimethoprim/sulfamethaxazole	0 (0.0)	0 (0.0)	106 (100)	≥ 16	11–15	≤ 10

**Fig. 1:**
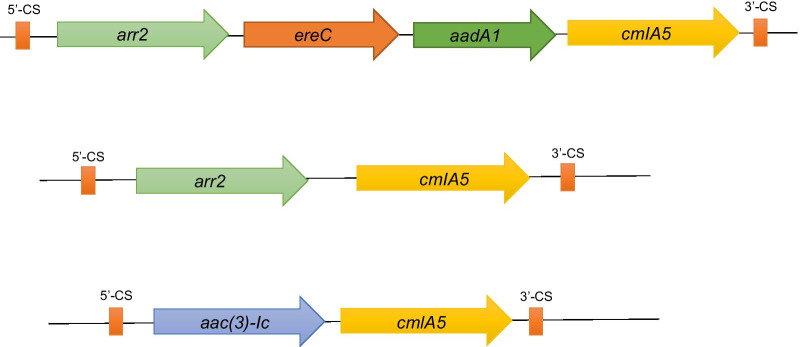
Genetic map of the three gene cassette arrangements found in the integron class 1 of *A. baumannii* strains. 5′-CS, 5′-conserved segment; 3′-CS, 3′-conserved segment; *arr2*, rifampin ADP-ribosylating transferase gene; *ereC*, erythromycin esterase gene; *aadA1*, aminoglycoside adenyltransferase A1 gene; *cmlA5*, chloramphenicol resistance protein A5 gene; *aac(3)-Ic*, aminoglycoside *N*-acetyltransferase AAC(3)-Ic gene

The presence of integrons class 1 was detected by amplification of integrase (*intI1*) in 62 (58.5%) of the *A. baumannii* isolates (Table 3). Of 62 integron class 1 -positive *A. baumannii* strains, 49 (79.0%) were identified with at least one gene cassette and 13 (21.0%) of these were identified as empty integrons without gene cassettes. Mapping of integrons revealed three different gene cassettes including: *arr2*, *cmlA5*, *qacE1* (2300 bp); *arr-2*, *ereC*, *aadA1*, *cmlA7*, *qacE1* (4800 bp); and *aac(3)-Ic*, *cmlA5* (2250 bp) (Fig. 1) which were identified in 44 (70.9%), 2 (3.2%) and 3 (4.8%) integron class 1 positive *A. baumannii* isolates respectively. Also the frequency rates of *bla*_VIM_, and *bla*_IMP_ among 106 *A. baumannii* isolates were 102 (96.2%) and 62 (58.5%) respectively and 60 (56.6%) carried both *bla*_VIM_*,* and *bla*_IMP_ genes. The association between *intI1* + *bla*_VIM_, and *intI1* + *bla*_IMP_ genes was identified among 58 (54.7%), and 42 (39.6%) respectively. Forty (37.7%) of 106 *A. baumannii* isolates, carried all three *intI1* + *bla*_VIM_ + *bla*_IMP_ genes. It was also shown that 44 (41.5%), isolates were *intI1* (−); *bla*_VIM_ (+) and 4 (3.8%) of which identified as *intI1* (+); *bla*_*VIM*_ (−) whereas 20 (18.9%) and 20 (18.9%) out of 106 *A. baumannii* isolates were *intI1* (−); *bla*_IMP_ (+) and *intI1* (+); *bla*_IMP_ (−) respectively (Table 3).

**Table 3:** Distribution of *bla*_VIM_, *bla*_IMP*,*_* intI1* and gene cassettes among* Acinetobacter baumannii* isolates (N = 106)

Strains, N (%)	Genes			
	*intI1*	CS	*bla*_VIM_	*bla*_IMP_
31 (29.2)	+	+	+	+
14 (13.2)	+	+	+	−
2 (1.9)	+	+	−	+
9 (8.5)	+	−	+	+
2 (1.9)	+	+	−	−
4 (3.8)	+	−	+	−
20 (18.9)	−	−	+	+
24 (22.6)	−	−	+	−

Interpretation of the results of statistical analysis showed there was no significant association between the presence of class 1 integrons and age groups (*P* = 0.55), and burn size (*P* = 0.52). However the presence of class 1 integrons were significantly higher in female than male patients (*P* = 0.017).

## Discussion

*Acinetobacter baumannii* is one of the most important pathogens leading to infections in burn patients. Control of *A. baumannii* infections in these patients is a major challenge due to the proliferation of MDR strains [25]. In the current study, all isolates were identified as MDR strains. Also in accordance with other studies, we found that all our *A. baumannii* strains were carbapenem resistant [26]. Similarly to our study, 94.5% of *A. baumannii* isolated from burn patients were resistant to carbapenems in the study by Pournajaf et al., [5], suggesting that the mentioned group of antibiotics is no longer suitable for the treatment of infections caused by this bacterium. Determination of resistance by MIC showed that all strains were sensitive to colistin. These data are in agreement with those of Tarashi et al., [27] and previous studies [17], and show that colistin is still an effective antibiotic against MDR *A. baumannii*. In our study, *bla*_VIM_ was identified as the most common gene encoding MBLs in the vast majority of isolates, followed by *bla*_IMP_. According to the literature, a wide distribution of VIM type metallo-β-lactamase has been reported at Middle East CRAB [22]. An association between class 1 integrons and MBLs genes, particularly *bla*_IMP_ and *bla*_VIM_, has been reported in other studies [17]. However, our findings showed that the *bla*_IMP_ and *bla*_VIM_ genes were not located on the class 1 integrons and no association was found between MBL genes and the presence of class 1 integrons among studied isolates. The reason could be that these genes may have been located on the other region of bacterial DNA.

The analysis of integrons content revealed that 79.0% of studied integron class 1 positive *A. baumannii* strains carried at least one gene cassette, while 21.0% had no cassettes and carried empty integrons. Considering that strains with empty integrons have the potential to capture cassettes carrying resistance genes, this result could be remarkable. The most common integron cassettes identified among integron-positive *A. baumannii* strains were *arr2*, *cmlA5, qacE1*, which encode rifampin ADP -ribosyltransferase, chloramphenicol transporter and quaternary ammonium resistance protein leading to resistance to rifampicin, chloramphenicol and quaternary ammonium compounds, respectively. Rifampin resistance due to the *arr-2* gene carried by class 1 integrons has been documented in *A. baumannii* strains [28]. Also other variants of *cmlA* gene have been reported in other studies and it seems that this is the first report of detecting *cmlA5* and *cmlA7* variants in *A. baumannii* strains in Iran [24]. Other cassettes, including *aadA1* and *aac(3)-Ic* encoding aminoglycoside adenylase and aminoglycoside acetyltransferase respectively were found to be less common in the present study. In researches conducted in other parts of the world, including Taiwan, mainland China, and France, different variants of the *aadA* gene such as *aadA1*, *aadA2* and *aadA6* have been identified in multidrug-resistant *A. baumannii* strains [23, 24, 29]. The identification of *aadA1* and *aac(3)-Ic* genes in cassettes of class 1 integrons could explain the high resistance to aminoglycosides in the present study. Despite the high resistance to antibiotics such as ceftazidime, ciprofloxacin, and trimethoprim/sulfamethaxazole, no gene cassette encoding resistance to these antibiotics was found in the present study. Therefore, the development of resistance to mentioned antibiotics may depend on the presence of genetic elements other than integrons.

## Limitations

This project is a cross-sectional study. However, to demonstrate the clinical relevance and dynamic community structure of clinical isolates, we need to continuously monitor the outbreaks using PFGE, MLST, or WGS tools. More information about resistant bacteria helps us to respond to how bacteria spread throughout clinical settings. Part of the limitations of the current study is also due to limited resources. However, other genes responsible for carbapenem resistance and MBL formation could be identified by WGS. In addition to class 1, detection of gene cassette arrays associated with other integron classes could level up the study. Seemingly, the data obtained in the present study may provide a basis for future studies and assess the trend of infection generated by *A. baumannii* in burn patients in our region.

## Conclusion

A high prevalence of MBLs genes especially *bla*_VIM_ was identified in studied MDR *A. baumannii* isolates. In addition, most of the VIM type MBL-positive strains carried class 1 integrons. Furthermore, the gene cassettes arrays of integrons including *cmlA5* and *cmlA7* were detected, for the first time, in *A. baumannii* strains in Iran.

## Data Availability

All data generated or analyzed during this study are included in this article. The class 1 integrons gene cassette arrays including *arr2*-*cm1A5*, *arr*-*2-ereC*-*aadA1*-*cmlA7*, and *aac(3)Ic*-*cmlA5* have been deposited in GenBank database under accession numbers MZ361740 (https://www.ncbi.nlm.nih.gov/nuccore/MZ361740), MZ361741 (https://www.ncbi.nlm.nih.gov/nuccore/MZ361741), and MZ361742 (https://www.ncbi.nlm.nih.gov/nuccore/MZ361742), respectively.

## Abbreviations

ADP: Adenosine diphosphate
ATCC: American type culture collection
BLAST: Basic local alignment search tool
CLSI: Clinical and laboratory standards institute
CRAB: Carbapenem-resistant *A. baumannii*
IMP: Imipenemase
MBLs: Metallo-β-lactamases
MDR: Multidrug-resistant
MICs: Minimum inhibitory concentrations
MLST: Multilocus sequence typing
NCBI: National center for biotechnology information
O/F: Oxidative/fermentative glucose
PBPs: Penicillin-binding proteins
PCR: Polymerase chain reaction
PDR: Pan-drug resistant
PFGE: Pulse field gel electrophoresis
TSB: Trypticase soy broth
TSI: Triple sugar iron
SPSS: Statistical package for the social sciences
VIM: Verona integron-encoded metallo-β-lactamases
WGS: Whole genome sequencing
XDR: Extensively-drug resistant
